# The Role of Congenital Cytomegalovirus Infection in Adverse Birth Outcomes: A Review of the Potential Mechanisms

**DOI:** 10.3390/v13010020

**Published:** 2020-12-24

**Authors:** Annete Njue, Carolyn Coyne, Andrea V. Margulis, Dai Wang, Morgan A. Marks, Kevin Russell, Rituparna Das, Anushua Sinha

**Affiliations:** 1 RTI Health Solutions, Manchester M20 2LS, UK; 2 Department of Pediatrics, University of Pittsburgh School of Medicine, Pittsburgh, PA 15213, USA; coynec2@pitt.edu; 3 RTI Health Solutions, 08028 Barcelona, Spain; amargulis@rti.org; 4 Merck & Co., Inc., Kenilworth, NJ 07033, USA; dai_wang@merck.com (D.W.); morgan.marks@merck.com (M.A.M.); kevin.russell@merck.com (K.R.); rituparna.das@merck.com (R.D.); anushua.sinha@merck.com (A.S.)

**Keywords:** cytomegalovirus (CMV), adverse birth outcome, IUGR, stillbirth, preterm birth, mechanism

## Abstract

Human cytomegalovirus (CMV) is a major cause of nonhereditary adverse birth outcomes, including hearing and visual loss, neurologic deficits, and intrauterine growth retardation (IUGR), and may contribute to outcomes such as stillbirth and preterm delivery. However, the mechanisms by which CMV could cause adverse birth outcomes are not fully understood. This study reviewed proposed mechanisms underlying the role of CMV in stillbirth, preterm birth, and IUGR. Targeted literature searches were performed in PubMed and Embase to identify relevant articles. Several potential mechanisms were identified from in vitro studies in which laboratory-adapted and low-passage strains of CMV and various human placental models were used. Potential mechanisms identified included impairment of trophoblast progenitor stem cell differentiation and function, impairment of extravillous trophoblast invasiveness, dysregulation of Wnt signaling pathways in cytotrophoblasts, tumor necrosis factor-α mediated apoptosis of trophoblasts, CMV-induced cytokine changes in the placenta, inhibition of indoleamine 2,3-dioxygenase activity, and downregulation of trophoblast class I major histocompatibility complex molecules. Inherent challenges for the field remain in the identification of suitable in vivo animal models. Nonetheless, we believe that our review provides useful insights into the mechanisms by which CMV impairs placental development and function and how these changes could result in adverse birth outcomes.

## 1. Introduction

Human cytomegalovirus (CMV) is a host-restricted, endemic, and ubiquitous member of the Herpesviridae family of viruses [1,2,3]. It has a large double-stranded DNA genome of 236 kb encoding at least 167 gene products, of which more than 40 are involved in modulating host immune responses following infection [1,4]. Human CMV establishes a lifelong, latent infection after primary infection, employing multiple mechanisms to evade the immune system [1]. Cytomegalovirus is the most common cause of congenital viral infection, with a prevalence at birth of 0.4–1% [5]. Globally, seroprevalence of CMV infection in women of reproductive age is estimated to be 86% and increases with age [6,7]. The effects of CMV infection on fetuses and newborns can include intrauterine growth retardation (IUGR) and auditory, visual, and other neurologic deficits; the most common sequela is sensorineural hearing loss [8,9,10].

Congenital CMV (cCMV) can arise from primary or nonprimary (reactivation of latent infection or reinfection with a new strain) maternal infection [11,12]. Congenital CMV infection can cause fetal injury through direct damage to the fetus and indirectly through placental dysfunction resulting from infection or immunity-mediated destruction, without evidence of transmission of the virus to the fetus [13,14,15,16]. Cytomegalovirus infects and/or bypasses the placenta before it infects the embryo or fetus and is thought to cause adverse pregnancy outcomes that are associated with placental pathology, including preterm birth and IUGR [17].

**Figure 1:**
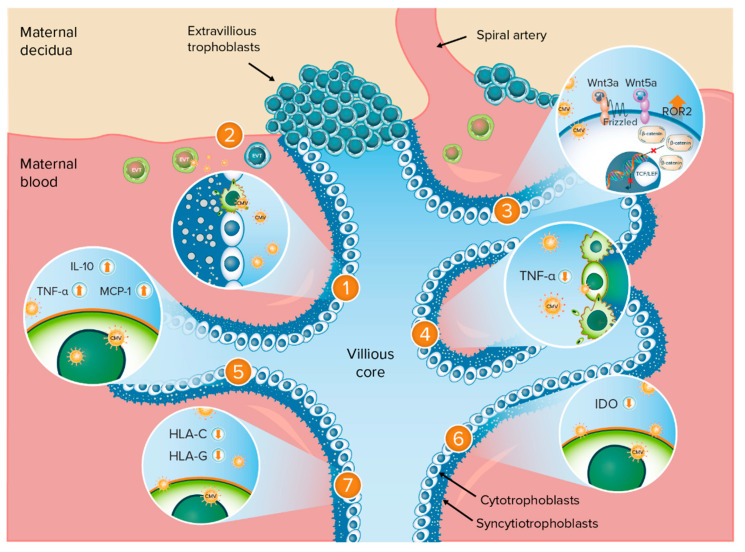
Anatomy of the maternal–fetal interface and potential mechanisms of adverse birth outcomes following CMV infection. Potential mechanisms of adverse birth outcomes following CMV infection: 1. Impairment of TBPC differentiation and function; 2. Impairment of EVT invasiveness; 3. Dysregulation of Wnt signaling pathways in cytotrophoblasts; 4. TNF-α-mediated apoptosis of trophoblasts; 5. CMV-induced cytokine changes in the placenta; 6. Inhibition of IDO activity; 7. Downregulation of trophoblast class I MHC molecules. CMV = cytomegalovirus; EVT = extravillous trophoblasts; HLA = human leukocyte antigen; IDO = indoleamine 2,3-dioxygenase; IL-10 = interleukin-10; MCP = monocyte chemoattractant protein; MHC = major histocompatibility complex; ROR2 = tyrosine kinase-like orphan receptor 2; TBPC = trophoblast progenitor stem cells; TCF/LEF = T-cell-specific factor/lymphoid enhancer-binding factor; TNF = tumor necrosis factor; Wnt = Wingless. Orange up-arrow indicates upregulation or increased expression; orange down-arrow indicates downregulation or inhibition.

Understanding the mechanisms by which CMV may cause adverse birth outcomes requires a thorough understanding of the anatomy of the maternal–fetal interface, presented in Figure 1. The placenta is composed of chorionic villi, where the cytotrophoblast stem cells or trophoblast progenitor stem cells (TBPCs) are found within the villous core [17,18]. These cells differentiate along two pathways into the trophoblast populations that comprise the floating and anchoring chorionic villi, which have different properties and functions and differentially interface with the maternal compartment. In all villi, cytotrophoblast cells fuse to form a multinucleated syncytiotrophoblast layer that covers the villus surfaces. These cells are in direct contact with maternal blood and are involved in transporting various substances (e.g., gas, nutrients, and waste) to and from the embryo or fetus given that maternal and fetal blood do not mix during pregnancy. In anchoring villi, extravillous trophoblasts (EVTs) remain as single cells that aggregate into columns and invade the uterine wall. Invasion by EVTs is essential for normal pregnancy. These cells not only physically anchor the fetal-derived placenta to the maternal decidua but also remodel the uterine vasculature, resulting in a hybrid vasculature composed of fetal and maternal cells that supply maternal blood to the placenta. The process of EVT-mediated remodeling occurs during the first trimester of human pregnancy, with maternal blood directly contacting only the placenta in the later stages of the second and third trimesters. Thus, human pregnancy can be divided into two distinct stages—one in which the placenta is not in contact with maternal blood (first trimester) and one in which it is (second and third trimesters).

There are currently no licensed preventive treatments for cCMV infection. The efficacy of antiviral treatment for cCMV disease is variable [19], and understanding the molecular biology of CMV infection may lead to the identification of future therapies. However, the mechanisms by which CMV causes adverse birth outcomes are not fully understood. The aim of this study was to review the proposed mechanisms underlying the role of CMV in adverse birth outcomes, including stillbirth, preterm birth, and IUGR, either via placental dysfunction or other mechanisms.

## 2. Materials and Methods

Targeted literature searches were performed in PubMed and Embase on 22 October 2018, and updated on 17 July 2020, with no date, language, or geographical restrictions. Search terms included combinations of free text and/or Medical Subject Headings (MeSH) for the health condition of interest (including CMV and cCMV) and outcomes of interest (including stillbirth, preterm birth, and IUGR) (Table 1). Bibliographies of literature identified in the database search were also reviewed. Comments, letters, case reports, and phase 1 clinical studies were excluded. Given the species specificity of CMV and differences in the structures of the human and animal placentas, animal studies were not included.

## 3. Results

### 3.1. Search Results

The literature review identified 15 articles describing potential mechanisms that may be associated with the adverse birth outcomes of interest. All 15 articles described in vitro studies using placental models. Placental cell lines or cultured cells were used in 13 of the identified studies, and 6 studies used explants (Table 2). Four studies used cells or tissues obtained from term placentas, and 10 studies used cells or tissues obtained from first- or second-trimester pregnancies. Laboratory-adapted strains of human CMV were used in eight studies, and low-passage CMV isolates were used in six studies (Table 2). The most commonly used laboratory strain was AD169 (seven studies), and the most commonly used low-passage strains were VR1814 and VHLE (three studies each). Laboratory strains are highly passaged and harbor mutations that make them better suited to in vitro conditions [20]. However, even low-passage strains can harbor clinically significant mutations [20]. The laboratory-adapted AD169 strain has acquired mutations in the UL128-131A locus, which affects growth properties and virus tropism, making it better adapted to grow in fibroblasts [20,21]. Low-passage strains such as VR1814 express the pentamer glycoprotein complex gH/gL/pUL128-131A, which plays an important role in virus entry into epithelial and endothelial cells [21]. The laboratory strain AD169 lacks this pentamer and does not infect epithelial and endothelial cells [21].

This review identified several mechanisms by which CMV could be linked to adverse birth outcomes (IUGR, stillbirth, or preterm birth); these are described in more detail in the following sections. These mechanisms are summarized in Figure 1.

### 3.2. Impairment of TBPC Differentiation and Function

Trophoblast progenitor stem cells, which are found in the core of chorionic villi, give rise to the mature cells of the human placenta, cytotrophoblasts, and multinucleated syncytiotrophoblasts (Figure 1) [25]. The continued replication, self-renewal, and differentiation of the multipotent TBPCs are essential for the formation of new chorionic villi [14].

Cultured TBPCs are susceptible to infection with both the low-passage VR1814 and the attenuated laboratory AD169 CMV strains, and both strains can replicate in these cells [21]. Infection of cultured TBPCs with these strains indicates that infection has the potential to impair villous growth and differentiation, thereby reducing the mature trophoblast populations (cytotrophoblasts and syncytiotrophoblasts) [21]. Infection of TBPCs could also promote viral spread among various cell types in the placenta [21].

Cytomegalovirus infection of TBPC cell lines dysregulates key proteins required for self-renewal and differentiation and inhibits normal division and development into mature placental cells, thereby interfering with the earliest steps in the growth of new villi [18]. As this affects the earliest stages of trophoblast development, this mechanism may disproportionately affect pregnancies earlier in gestation when the villi are maturing. In vitro studies using TBPC cultures infected with the low-passage CMV strain VR1814 revealed that CMV was able to replicate in these cells and alter the expression or subcellular localization of proteins required for cell cycle progression, pluripotency, differentiation, and invasiveness (Table 3) [18].

### 3.3. Impairment of EVT Invasiveness

Cytomegalovirus replicates in EVTs and impairs their ability to differentiate and invade by altering the expression of key molecules [34]. Reduced EVT invasion could have several deleterious impacts on pregnancy, such as impairing remodeling of the uterine vasculature and restriction of maternal blood flow and access of the fetus to nutrients, leading to IUGR and miscarriage [34,35].

Matrix metalloproteinases (MMPs) 2 (MMP-2) and 9 (MMP-9) are degradative enzymes expressed in cytotrophoblasts that are involved in EVT migration and invasiveness, which are critical to successful pregnancy [24,25]. Matrix metalloproteinase-9 is expressed in early gestation and midgestation; levels decrease when invasion is complete [24,25]. Matrix metalloproteinase-2 is expressed throughout gestation, although it is active in early gestation and midgestation [24]. The oncogene c-erbB-2 is also highly expressed in cytotrophoblasts and is involved in cytotrophoblast migration and invasiveness [24]. Extravillous trophoblasts also express adhesion molecules (e.g., α1β1 integrin, which is also needed for invasion), and the cytokine interleukin-10 (IL-10) [17,36].

Cytomegalovirus infection has been shown to downregulate MMP activity directly and indirectly through the expression of CMV IL-10 and the upregulation of IL-10 [24,25]. This suggests that CMV IL-10 can alter proteinase activity and impair extracellular matrix degradation and cytotrophoblast function via a paracrine mechanism. In vitro studies showed that MMP-2, MMP-9, and c-erbB-2 expression levels were significantly lower in early pregnancy placental villous explants infected with the CMV laboratory strain AD169 than in uninfected explants [24]. An earlier study suggested that CMV reduced cytotrophoblast invasiveness through autocrine effects that increase CMV IL-10 [25]. In this study, the low-passage CMV strain VR1814 downregulated MMP-9 activity in cultured cytotrophoblasts. Infected cytotrophoblasts produced CMV IL-10, which upregulated human IL-10. Both CMV IL-10 and human IL-10 impaired cytotrophoblast migration in vitro.

Cytomegalovirus infection downregulates the expression of the adhesion molecule α1β1 integrin in EVTs, thereby impairing their invasiveness [17]. This was demonstrated in a study in which cytotrophoblast cultures infected with the CMV laboratory strain AD169 were found not to express α1β1 integrin [17]. This study also showed that CMV infection dramatically impaired cytotrophoblast invasiveness in cytotrophoblast cultures when compared with uninfected cytotrophoblast cultures.

Peroxisome proliferator-activated receptor γ (PPARγ) activation is a key effector of CMV infection in cytotrophoblasts and is thought to be involved in the pathophysiology of IUGR [22,23]. Peroxisome proliferator-activated receptor γ is a ligand-activated nuclear receptor that is essential in placentation and trophoblast function [22]; it regulates lipid metabolism, inflammation, and the immune response and controls the invasiveness and differentiation of trophoblasts. Cytomegalovirus infection with low-passage strain VHLE has been shown to induce the activation of PPARγ in cytotrophoblast culture and an immortalized cell line (human invasive proliferative extravillous cytotrophoblast (HIPEC)), leading to impairment of migration and invasiveness [23].

Activation of PPARγ in CMV-infected trophoblasts is mediated by two natural ligands, namely 15-hydroxyeicosatetraenoic acid (15-HETE) and 13-hydroxyoctadecadienoic acid (13-HODE) [22]. These ligands are secreted by EVTs (HIPEC) and first-trimester placental explants infected with the laboratory strain AD169 and low-passage VHLE CMV strain, respectively [22]. Treatment of CMV-infected (AD169 laboratory strain) HIPEC cells with 13-HODE or 15-HETE significantly impaired the migratory properties of these cells when compared with uninfected, untreated HIPEC cells [22].

Thus, in EVTs, CMV infection might activate PPARγ and reduce the expression of α1β1 integrin, c-erbB-2, and MMPs, thereby impairing the ability of these cells to invade the uterine vasculature. Infected cells increase the production of immunosuppressive human and CMV IL-10, which further reduce invasiveness. Migration and invasiveness of EVTs are essential for implantation, vascular remodeling, and fetal growth. As such, dysregulation of key molecules involved in migration or invasion due to CMV infection could adversely affect placental development and consequently the outcome of pregnancy, leading to fetal loss or restricted fetal growth [17,23,25]. However, the exact consequences are not known and are likely to vary depending on gestational age [17]. Infection of trophoblasts soon after implantation may interfere with implantation, leading to early pregnancy loss. Infection at a later stage could impair the formation of both floating and anchoring villi. Impairment of floating villi may result in a reduction in the surface area of the villi, leading to fetal growth restriction. Impairment of anchoring villi may affect EVT invasion and attachment of placental cell columns to the uterus, changes that may be associated with preterm labor [17].

Table 4 summarizes the impact of CMV infection on the expression of molecules involved in cytotrophoblast differentiation and invasiveness.

### 3.4. Dysregulation of Wnt Signaling Pathways in Cytotrophoblasts

The Wingless (Wnt) signaling pathway plays an important role in the differentiation and migration of cytotrophoblasts and EVTs [37]. Wnt ligands bind to various receptors to activate different downstream effector pathways, including the canonical or Wnt-β-catenin-dependent pathway and the noncanonical or β-catenin-independent pathway. Wnt signaling pathways are involved in a diverse range of biological functions, including development and homeostasis, and are tightly regulated during development [26,38]. At least 19 different Wnt ligands and 10 transmembrane receptors have been identified in humans [37]. Cytomegalovirus infection might thus alter trophoblast migration by inhibiting both noncanonical and canonical Wnt signaling [16,26].

The major effector protein in the canonical Wnt signaling pathway is β-catenin, which is normally retained in the cytoplasm in an inactive state through its interaction with a multiprotein complex [26,39]. Binding of Wnt ligands to the transmembrane receptor results in the release of β-catenin and subsequent translocation into the nucleus, where it promotes the transcription of Wnt target genes upon binding to its receptor [39]. Cytomegalovirus infection using the laboratory Towne strain has been shown to induce the sequestration and degradation of the β-catenin protein in EVTs (SGHPL-4 cell line), preventing its downstream signaling activities [26]. Cytomegalovirus infection also inhibited migration of EVTs and resulted in a significant reduction in MMP-2 and MMP-9 expression compared with uninfected EVTs [26].

Cytomegalovirus infection also modulates the expression of noncanonical Wnt receptor tyrosine kinase-like orphan receptor 2 (ROR2) to alter Wnt5a-mediated signaling and inhibit trophoblast motility [16]. Wnt5a binds to ROR2, leading to activation of the Wnt-Ca2+ noncanonical Wnt pathway [16]. Compared with uninfected cells, the infection of human EVTs (SGHPL-4 cell line) with the CMV laboratory strain AD169 significantly inhibited Wnt-5a-mediated trophoblast migration and significantly increased ROR2 receptor expression: this led to a significant reduction in Wnt5a-induced trophoblast migration, resulting in a reduction in trophoblast motility (suggesting that ROR2 plays a critical part in the effect of CMV on trophoblast migration) [16]. Increased ROR2 receptor expression also led to impairment of Wnt3a activity, resulting in reduced trophoblast motility (suggesting that ROR2 may be involved in antagonizing canonical Wnt signaling in trophoblasts, resulting in a reduction in trophoblast motility) [16]. Inhibition of CMV-induced altered expression of ROR2 partially restored CMV-induced trophoblast migration [16].

These results suggest that noncanonical Wnt5a signaling and canonical Wnt3a signaling might be involved in the molecular mechanisms underlying CMV-induced reduction of trophoblast motility. Inadequate trophoblast invasion and migration results in abnormal placentation, which is associated with IUGR, stillbirth, and preterm birth, all of which are adverse pregnancy outcomes associated with CMV infection [16].

### 3.5. Trophoblast Apoptosis Mediated by Tumor Necrosis Factor α

Villous trophoblast turnover is a normal event in placental development; however, it is increased in pregnancies complicated by IUGR, suggesting that cell death signaling might be involved in the pathophysiologic mechanisms of IUGR [27,29]. Cytomegalovirus infection is associated with IUGR, even in the absence of fetal infection, suggesting that placental dysfunction may be involved [29].

In in vitro studies, CMV infection of villous cytotrophoblasts has been shown to cause a rapid loss of neighboring trophoblasts through apoptosis mediated by tumor necrosis factor α (TNF-α) secretion [27]. Cytomegalovirus infection of primary cytotrophoblast or syncytiotrophoblast cultures obtained from normal full-term placentas using the laboratory strain AD169 resulted in the loss of half of the cells within 24 h of virus challenge. Cytomegalovirus-infected cells produced TNF-α in response to infection, likely via innate immune signaling; this cytokine induced apoptosis in uninfected cells through paracrine-mediated effects. Treatment of CMV-infected cell cultures with anti-TNF-α antibody and epidermal growth factor completely inhibited CMV infection-induced trophoblast apoptosis and cell loss. This CMV-induced loss of trophoblasts may lead to the placental villitis seen in CMV-infected placentas. Thus, CMV infection may damage the placental trophoblast barrier by accelerating trophoblast turnover and reducing their capacity for renewal [27].

Further study suggests that ultraviolet-inactivated CMV may induce syncytiotrophoblast apoptosis, and this may be mediated through the interaction of inactivated CMV with toll-like receptor 2 (TLR2) on syncytiotrophoblasts, leading to TNF-α secretion [28]. Ultraviolet-inactivated CMV strains AD169 and a low-passage variant Kp7 induced similar levels of apoptosis in cultured syncytiotrophoblasts (but not cultured cytotrophoblasts). Ultraviolet-inactivated CMV activated TLR2, which led to the secretion of TNF-α in syncytiotrophoblast cultures; neutralizing the antibody to TLR2 inhibited ultraviolet-inactivated, CMV-induced, TNF-α expression and consequently inhibited the increase in syncytiotrophoblast apoptosis. However, the changes observed were modest, and it is unclear how CMV would induce syncytiotrophoblast apoptosis.

### 3.6. CMV-Induced Cytokine Changes in the Placenta

During pregnancy, cytokines are involved in regulating interactions between the placenta and the maternal immune system [15]. Cytokines directly influence placental development and function, including the growth of anchoring and floating villi, which are essential for the delivery of oxygen and nutrients to the developing fetus. Cytomegalovirus has immunomodulatory properties that alter the host immune response to infection and has been shown to alter cytokine profiles in the placenta [15], changes that could lead to adverse birth outcomes such as IUGR, pregnancy loss, and preterm delivery.

As noted previously, human and CMV IL-10 expression is upregulated in CMV infection. Other cytokines and chemokines that are upregulated during CMV infection of trophoblasts include TNF-α and monocyte chemoattractant protein-1 (MCP-1). The chemokine MCP-1 and cytokine TNF-α have strong proinflammatory effects and are involved in the regulation of placental development and function, maintenance of pregnancy, and protection against pathogens. Expression of MCP-1 and TNF-α is elevated in CMV-infected placentas from stillbirths: MCP-1 and TNF-α protein localized in syncytiotrophoblasts, cytotrophoblasts, mesenchymal, and endothelial cells of chorionic villi in CMV-infected placentas and uninfected placentas from stillbirths [15]. Little is known about the effects of cytokines on the local T cell responses at the fetal–maternal interface. Overactive inflammatory responses may break fetal–maternal tolerance and lead to placenta damage.

Increased expression of MCP-1 and TNF-α has also been observed in placental villous explants infected with the CMV AD169 strain compared with uninfected explants [15]. Explant infection with the Merlin strain resulted in increased expression of MCP-1 and TNF-α; however, the difference was not significant compared with uninfected explants. Cytokines localized in the chorionic villi of the placental explants in a manner similar to localization seen in clinically infected and uninfected placental tissue from stillbirths.

Elevated MCP-1 expression is associated with adverse pregnancy outcomes, including IUGR, spontaneous abortion, pregnancy loss, and preterm delivery, all of which are associated with cCMV infection [15]. Elevated TNF-α expression is associated with spontaneous abortion and preterm delivery. According to the authors, these cytokines could be used as markers of adverse pregnancy outcomes due to CMV infection and potentially as therapeutic targets [15]. More studies are needed to confirm the association.

### 3.7. Inhibition of Indoleamine 2,3-Dioxygenase Activity

Indoleamine 2,3-dioxygenase (IDO) is highly expressed in the placenta and is thought to be involved in maintaining maternal–fetal tolerance, preventing maternal immune rejection of the fetus through suppression of maternal T-lymphocytes [30]. In first-trimester placentae, IDO is expressed in stromal cells; in term placentae, IDO is expressed in trophoblasts and endothelial cells [30]. Inhibition of IDO activity by CMV infection inhibits control of the maternal immune response and may be a possible mechanism for fetal loss (miscarriage or stillbirth) and preterm birth [30].

In vitro studies have shown that the constitutive activity of IDO is higher in early (first trimester) than in term placental explants [30]. Both constitutive and interferon gamma (IFNγ)-induced IDO activity are impaired in CMV-infected placentae. Infection of early placental cultures with CMV strain VHLE resulted in suppression of constitutive IDO activity compared with uninfected cultures. In contrast, constitutive IDO activity remained unchanged in CMV-infected term placentae compared with uninfected controls. Infection of early and term placental cultures resulted in suppression of IFNγ-induced IDO expression.

### 3.8. Downregulation of Trophoblast Class I Major Histocompatibility Complex Molecules

Maternal–fetal tolerance involves the expression of classical human leukocyte antigen (HLA) class I major histocompatibility complex (MHC) on the surface of cytotrophoblasts, which are in direct contact with the maternal immune system at the placental–uterine interface [31,40]. Cytotrophoblasts express both the classical class I molecule HLA-C and the nonclassical class I MHC molecule HLA-G [31].

In vitro studies have shown that CMV gene products US3 and US6 downregulate the expression of HLA-C and HLA-G, which are thought to be involved in protecting the fetus from rejection [31]. These CMV gene products can independently reduce class 1 H chain expression on the cell surface. A study using a human-trophoblast-derived cell line (JEG-3) stably transfected with the human class I genes showed that CMV US3 and US6 downregulated the cell surface expression of both HLA-G and HLA-C by two different mechanisms. Cytomegalovirus US3 was shown to bind to HLA-G and HLA-C, retaining them in the endoplasmic reticulum. In contrast, CMV US6 was shown to inhibit the transporter associated with antigen-processing-mediated peptide translocation from the cytosol to the endoplasmic reticulum. Despite the different mechanisms, the effect is the same: downregulation of MHC class 1 expression (HLA-C and HLA-G) on the trophoblast cell surface. It is possible that other CMV gene products may also be involved in downregulating the expression of MHC class 1 molecules. The downregulation of these molecules following CMV infection may result in cellular death mediated by maternal natural killer cells, which may lead to spontaneous fetal loss [31].

## 4. Discussion

The aim of this review was to evaluate the mechanisms by which CMV could be linked to adverse birth outcomes (IUGR, stillbirth, or preterm birth). Multiple potential mechanisms were identified from this comprehensive evaluation of the literature, including impairment of TBPC differentiation and function, impairment of EVT invasiveness, dysregulation of Wnt signaling pathways in cytotrophoblasts, TNF-α mediated apoptosis of trophoblasts, CMV-induced cytokine changes in the placenta, inhibition of IDO activity, and downregulation of trophoblast class I MHC molecules.

Results from in vitro studies show that CMV infection dysregulates key proteins required for self-renewal and differentiation of TBPCs and inhibits development into mature placental cells [18]. Impairment of TBPC differentiation by CMV infection could interfere with the growth of new villi, contributing to impaired placental development that could lead to IUGR [18]. Assessment of placentas from cases of IUGR with primary CMV infection shows unusual clusters or “islands” of EVTs, which suggests arrested differentiation [41]. Taken together, the in vitro study results demonstrating CMV’s interference with progenitor cell differentiation and the pathological findings of arrested cytotrophoblast differentiation in placentas from CMV-positive IUGR cases provide evidence of the molecular mechanisms underlying IUGR [18].

Other mechanisms by which CMV could lead to abnormal placental development and adverse birth outcomes are via immune-mediated mechanisms and placental cell apoptosis. Cytomegalovirus reduces the expression of IDO and MHC class I proteins HLA-C and HLA-G [30,31]. Given the role of these molecules in fetal tolerance, CMV infection could disrupt this protective mechanism, potentially leading to fetal loss and preterm birth [30,31]. Cytomegalovirus infection also increases the loss of trophoblasts through apoptosis mediated by TNF-α secretion, which could lead to placental villitis and IUGR [27,28,29].

Given the complexities associated with studying the impact of CMV infection on the human placenta, whether the mechanisms identified translate to human pregnancy require further study. All studies included in this review were in vitro studies in which various human placental models were used, including early and term cell cultures of primary placental cells (e.g., EVTs), immortalized cell lines (e.g., SGHPL-4 (trophoblast), JEG-3 (trophoblast), and HIPEC (EVT)), and explants. The advantage of cell lines is their ability to replicate rapidly in culture [42]. Although primary placental cells can be isolated from early and term placentas, single-cell populations do not represent the different cell interactions, and multiple regulators present in the in vivo placenta [42]. Furthermore, cell lines reflect only a part of the cellular heterogeneity of the placental trophoblast, which is essential to the normal physiology of the placenta [42]. Thus, data from studies using cultured cells and cell lines may not reflect the in vivo situation. Explants have an intact microarchitecture, allowing cell–cell interactions and paracrine communications to be maintained [42]. As such, studies using explants provide data that could be more relevant to the in vivo situation than data obtained from primary placental cell cultures or cell lines [43].

The results of the studies identified in this review provide useful insights into the mechanisms by which CMV impairs placental development and function and how these changes could be linked to adverse birth outcomes such as IUGR, preterm birth, and stillbirth. Cytomegalovirus infection may cause placenta damage directly through the viral cytopathic effect or via excessive immune defense. Although not much is known about the immune responses elicited by CMV infection in the placenta, it is conceivable that hyperactive inflammatory innate response or dysregulated adaptive host immune defense may lead to tissue damage [13,44]. Mechanisms that affect trophoblastic invasion of the decidua would be more relevant in early pregnancy (as the placenta forms) than in late pregnancy; however, it is likely that that impairment of differentiation may be a problem throughout pregnancy. Although a variety of mechanisms by which CMV affects the placenta was described in the identified studies, a consensus on the association of mechanisms described with adverse birth outcomes has not emerged. This is complicated by the lack of in vitro and in vivo models that fully recapitulate the human maternal–fetal interface and the restricted species tropism of human CMV.

Although nonhuman primate models that better reflect the human placental interface have emerged as valuable tools to study a variety of aspects of CMV vertical transmission [45], the associated costs and difficulty of establishing such models preclude their wide use. The species specificity of CMV imposes a challenge in defining a suitable animal model for human CMV [35,36,46]. Nonhuman primate CMVs are the most closely related to human CMV [47]. Rhesus CMV bears numerous genomic and functional similarities to human CMV, with similar pathogenesis in rhesus and human cCMV infections [47]. Nonhuman primates are widely used in biomedical research in the United States (US), with the National Institutes of Health (NIH) funding many of these studies [48]. However, the 2020 US spending bill signed in December 2019 restricts some animal research and requires the NIH to explore alternatives to the use of nonhuman primates [49]. Government policy issued in 2019 also restricts the use of human fetal tissue obtained from elective terminations in all extramurally and intramurally funded grants [50]. Thus, researchers in the US are no longer able to collect first- and second-trimester tissue for these studies.

This study has several limitations. Most of the identified studies used cultured cells, and the results may not be applicable to the clinical situation. Although the literature search was broad and reference lists of included studies were reviewed for relevant publications, it is likely that additional mechanisms involved in adverse birth outcomes exist. Among these are the Hofbauer cells, which are the specialized fetal-derived macrophages found in the core of the chorionic villi [36]. These cells are susceptible to CMV infection and have been found to contain high amounts of virus in cases of cCMV in humans [51,52]. However, their role in the mechanism of CMV-associated adverse birth outcomes is largely unknown.

Another limitation is that the studies identified described mechanisms related to the placenta based on placental models; the review did not identify mechanisms related directly to fetal infection or fetal injury. The included studies did not provide direct clinical or pathological evidence linking the described mechanisms with the study outcomes involving wild-type strains that are present in the community. Included studies did not provide evidence of how the timing of CMV infection might affect outcomes and which mechanisms are more relevant in pregnancy as it progresses.

Understanding the molecular mechanisms of CMV infection as it affects the placenta, and the fetus is essential for identifying potential therapeutic and vaccine targets [16]. The results of the studies identified in this review provide insights into the potential mechanisms by which CMV impairs placental function. It is not known whether these potential mechanisms occur during human pregnancy as described in in vitro studies. However, we believe that our review provides useful insights into the mechanisms by which CMV impairs placental development and function and how these changes could result in adverse birth outcomes.

## Figures and Tables

**Table 1:** PubMed search terms.

Search No.	Search Terms
**Disease area**	
#1	Cytomegalovirus [Mesh]
#2	Cytomegalovirus [All fields]
#3	“Congenital cytomegalovirus” [All fields] OR (congenital [All fields] AND cytomegalovirus [All fields])
#4	“congenital cytomegalovirus infection” [All fields]
#5	“congenital cytomegalovirus disease” [All fields]
#6	“cCMV” [All fields] OR “CMV” [All fields]
#7	#1 OR #2 OR #3 OR #4 OR #5 OR #6
**Population**	
#8	Stillbirth [Mesh] OR Stillbirth [All fields] OR “Fetal Death” [Mesh] OR “Fetal Death” [All fields] OR “Intrauterine fetal death” [All fields] OR “Intrauterine foetal death” [All fields] OR “IUFD” [All fields] OR “adverse pregnancy outcomes” [All fields] OR “negative pregnancy outcomes” [All fields] OR “negative birth outcomes” [All fields] OR “spontaneous abortion” [All fields] OR “Pregnancy Complications, Infectious/drug therapy” [Majr]
#9	#7 AND #8
#10	“Premature birth” [Mesh] OR “Premature birth” [All fields] OR “Preterm birth” [All fields] OR “Preterm labor” [All fields] OR “Preterm labour” [All fields] OR “Premature labor” [All fields] OR “Premature labour” [All fields]
#11	#7 AND #10
#12	“Fetal Growth Retardation” [Mesh] OR “Fetal Growth Retardation” [All fields] OR “Intrauterine growth retardation” [All fields] OR “Intrauterine growth restriction” [All fields] OR “Fetal growth restriction” [All fields] OR “Foetal growth restriction” [All fields] OR “FGR” [All fields] OR “IUGR” [All fields] OR “Infant, Small for Gestational Age” [Mesh] OR “small for gestational age” [All fields]
#13	#7 AND #12
**Exclusion terms**	
#14	“Animals” [Mesh] NOT “Humans” [Mesh]
#15	“Comment” [Publication Type] OR “Letter” [Publication Type] OR “Editorial” [Publication Type] OR “Case Reports” [Publication Type] OR “Clinical Trial, Phase I” [Publication Type] OR “case study” [Title] OR “case studies” [Title] OR “case report” [Title] OR “case reports” [Title] OR “case series” [Title]
**Relevant studies**	
#16	(#9 OR #11 OR #13) NOT (#14 OR #15)

**Table 2:** Placental models and CMV strains used in the identified studies.

Author	Placental Model	CMV Strains
**Studies assessing TBPC differentiation and development**		
Tabata et al. [18]	▪ TBPC lines (isolated from human chorionic membranes (7.3 and 15.6 WG))	▪ VR1814 (low-passage strain)
Zydek et al. [21]	▪ TBPCs (established from first- and second-trimester placentas)	▪ VR1814 (low-passage strain) ▪ AD169 (lab strain)
**Studies assessing impairment of EVT invasiveness**		
Fisher et al. [17]	▪ Anchoring villi explant cultures (from 6- to 8-week (first trimester) human placentas obtained from donors with normal pregnancies prior to termination) ▪ Cytotrophoblasts (obtained from 10- to 16-week placentas)	▪ AD169 mutants RV798 and RV670 ^a^ (lab strain)
Leghmar et al. [22]	▪ Human immortalized extravillous cytotrophoblasts (HIPEC) ^b^ ▪ Placenta explants (from first-trimester placenta (8- to 11-week amenorrhea) from healthy women undergoing vaginal elective termination of pregnancy)	▪ AD169 (lab strain) ▪ VHLE (low-passage strain [<8 from parent stock])
Rauwel et al. [23]	▪ EVTs (obtained from chorionic villi from first-trimester placentas (8 to 12 WG) undergoing elective termination) and EVT-derived cell line (HIPEC)	▪ AD169 (lab strain) ▪ Towne (lab strain) ▪ VHLE (low-passage strain)
Tao et al. [24]	▪ Early pregnancy placental villous explants cultured in vitro (villus tissue was obtained from healthy pregnant women at 5–10 WG having voluntary abortion)	▪ AD169 (lab strain)
Yamamoto-Tabata et al. [25]	▪ Cytotrophoblasts (isolated from 10- to 16-week placentas)	▪ VR1814 (low-passage strain) ▪ AD169 (lab strain)
**Studies assessing Wnt signaling**		
Angelova et al. [26]	▪ Human extravillous cytotrophoblast cell line SGHPL-4 cells (derived from first-trimester chorionic villous tissue; exhibits features of invasive cytotrophoblasts, such as expression of HLA-G, CD9, and cytokeratin-7)	▪ Towne (lab strain) ▪ TR (BAC-derived low-passage strain)
van Zuylen et al. [16]	▪ Human EVT cell line SGHPL-4 (derived from first-trimester chorionic villous tissue)	▪ AD169 (lab strain)
**Apoptosis studies**		
Chan et al. [27]	▪ Human term villous cytotrophoblasts isolated from placentas obtained after normal term delivery or elective CS from uncomplicated pregnancies	▪ AD169 (lab strain)
Chan and Guilbert [28]	▪ Human term villous cytotrophoblasts isolated from placentas obtained after normal term delivery or elective CS from uncomplicated pregnancies	▪ AD169 (lab strain) ▪ Kp7 (low-passage strain) ▪ pUL32-EGFP human CMV (recombinant strain) ^c^
Chaudhuri et al. [29]	▪ Floating villi dissected from 28- to 34-week placentas ▪ Cultured trophoblasts	▪ AD169 (lab strain)
**Study assessing cytokine changes**		
Hamilton et al. [15]	▪ Placental villous explant histocultures (term placentas were collected from women undergoing elective CS who had a healthy pregnancy and were not in labor)	▪ Merlin (BAC-derived “wild-type” ^d^) ▪ AD169 (lab strain)
**IDO activity study**		
Lopez et al. [30]	▪ Early and term placenta tissue explants (from first-trimester placentas obtained following elective abortion (early) and normal term placentas obtained immediately after CS (term))	▪ VHLE (low-passage strain)
**MHC study**		
Jun et al. [31]	▪ Human-trophoblast-derived choriocarcinoma cell line JEG-3	▪ CMV gene products (cloned cDNAs ^e^): US2, US3, US6, US11, and ICP47

BAC = bacterial artificial chromosome; cDNA = complementary deoxyribonucleic acid; CMV = cytomegalovirus; CS = cesarean section; EVT = extravillous trophoblast; HIPEC = human invasive proliferative extravillous cytotrophoblast; HLA-G = human leukocyte antigen; IDO = indoleamine 2,3-dioxygenase; lab = laboratory; MHC = major histocompatibility complex; TBPC = trophoblast progenitor cells; WG = weeks of gestation. ^a^ With deletions in genes that downregulate the expression of classical MHC class I. ^b^ HIPECs are immortalized human EVTs that likely bear transcription or genomic alterations [22]. ^c^ Recombinant human CMV strain expressing enhanced green fluorescent protein (EGFP) fused to the capsid-associated tegument protein pUL32. ^d^ Although described as “wild-type” (Merlin (UL128+, RL13−)), this strain harbors RL13 mutations. ^e^ cDNA is DNA synthesized from a single-stranded RNA (e.g., messenger RNA (mRNA) or microRNA) template in a reaction catalyzed by the enzyme reverse transcriptase).

**Table 3:** Cytomegalovirus infection of TBPCs alters expression of key regulatory proteins.

Protein	Function	Impact of CMV Infection of TBPCs on Protein Expression or Localization
Geminin	▪ Involved in the maintenance of the undifferentiated state of neural progenitor cells ▪ Overexpression of geminin prevents neuronal differentiation	Upregulated
HMGA2	▪ Stem cell self-renewal factor	Downregulated ^a^
SOX2	▪ Stem cell self-renewal factor	Downregulated ^a^
GATA3	▪ Involved in trophoblast migration and invasion	Altered subcellular localization ^b^: ▪ Controls: nucleus ▪ Infected cells: cytoplasm
GATA4	▪ Differentiation-related transcription factor	Downregulated
Hand1	▪ Promotes trophoblast differentiation	Altered subcellular localization ^b^: ▪ Controls: nucleus ▪ Infected cells: cytoplasm
PPARγ	▪ Inhibits invasion ▪ Involved in the pathophysiology of IUGR	Upregulated
hCG	▪ Secreted from syncytiotrophoblasts and further stimulates syncytiotrophoblast formation ▪ Disturbance of syncytiotrophoblast maturation and function is found in IUGR	Downregulated ^c^

CMV = cytomegalovirus; hCG = human chorionic gonadotrophin; HMGA2 = high-mobility group AT-hook 2; IUGR = intrauterine growth retardation; PPARγ = peroxisome proliferator-activated receptor γ; TBPC = trophoblast progenitor cell. ^a^ Suggesting reduced capacity for self-renewal and pluripotency. ^b^ Loss of nuclear localization could further reduce the capacity of TBPCs to differentiate. ^c^ Suggesting reduced syncytiotrophoblast formation. Sources: Tabata et al. [18], Hughes et al. [32], Lee et al. [33].

**Table 4:** Cytomegalovirus infection: altered expression of molecules involved in cytotrophoblast differentiation and invasion.

Protein	Function	Impact of CMV Infection
MMP-2 and MMP-9	▪ MMP is one of the key enzymes that mediate cytotrophoblast migration and invasion (mainly MMP-2 and MMP-9)	▪ MMP-2 and MMP-9 expression levels significantly decreased in AD169-infected early pregnancy placental villous explants ▪ VR1814 (low-passage strain), but not AD169 (lab strain), reduced MMP-9 activity in UtMEC and differentiating-invading cytotrophoblasts
c-erbB-2	▪ Embryogenesis, tissue, repair, and regeneration ▪ Highly expressed in EVTs and is involved in invasion, which is key to successful pregnancy	▪ c-erbB-2 ^a^ protein expression level significantly decreased in AD169-infected early pregnancy placental villous explants
CMV IL-10 and human IL-10	▪ Pleiotropic cytokine (functions include downregulation of MMP activity)	▪ Infected cytotrophoblasts produced CMV IL-10, which upregulated human IL-10 ▪ Both CMV IL-10 and human IL-10 downregulated MMP activity, leading to impairment of cytotrophoblast migration in vitro
α1β1 integrin	▪ Adhesion molecule: expression is “switched on” by invading cytotrophoblasts in the anchoring villi; α1β1 integrin is needed for invasion	▪ Downregulated in cytotrophoblast cultures infected with AD169
PPARγ	▪ Regulates lipid metabolism, inflammation, and the immune response ▪ Controls trophoblast invasiveness and differentiation	▪ CMV infection (VHLE strain) induced PPARγ activity in infected U373MG astrocytoma cells in vitro and impaired cytotrophoblast migration and invasiveness in vitro

CMV = cytomegalovirus; EVT = extravillous trophoblast; IL-10 = interleukin 10; MMP = matrix metalloprotease; MMP-2 = MMP 2; MMP-9 = MMP 9; PPARγ = peroxisome proliferator-activated receptor γ; UtMEC = uterine myometrium endothelial cells. ^a^ Part of epidermal growth factor receptor, being a kind of oncogene protein with tyrosine kinase activity and closely linked with embryogenesis, tissue repair, and regeneration. Sources: Fisher et al. [17], Yamamoto-Tabata et al. [25], Pereira and Maidji [34], Tao et al. [24], Rauwel et al. [23].
